# A dose-response meta-analysis of the association between the maternal omega-3 long-chain polyunsaturated fatty acids supplement and risk of asthma/wheeze in offspring

**DOI:** 10.1186/s12887-022-03421-z

**Published:** 2022-07-16

**Authors:** Yin Jia, Yafang Huang, Huili Wang, Haili Jiang

**Affiliations:** 1 School of General Practice and Continuing Education, Capital Medical University, No. 10 You An Men Wai Xi Tou Tiao, Beijing, 100069 China; 2 Obstetrics Department, Beijing Obstetrics and Gynecology Hospital, Capital Medical University, Beijing Maternal and Child Health Care Hospital, No. 251 Yaojiayuan Road, Chaoyang District, Beijing, 100026 China

**Keywords:** Omega-3, Fish oils, Docosahexaenoic acids, Eicosapentaenoic acid, Randomized controlled trial, Prevention, Asthma, Wheeze, Offspring

## Abstract

**Background:**

Prenatal exposure to omega-3 long-chain polyunsaturated fatty acids (n-3 LC-PUFA) in oily fish may prevent asthma or wheeze in childhood.

**Objective:**

By limiting n-3 LC-PUFA capsules interventions commenced in pregnancy, this systematic review aimed to find more clear evidence on the relationship between the supplement with n-3 LC-PUFA during pregnancy and the risk of asthma/wheeze in offspring and to improve the life satisfaction of children with asthma.

**Methods:**

The Cochrane library, Embase, Medline, Web of Science, and PubMed were searched from origin to March 2021 in the above-mentioned databases. Studies selection, data of characteristics extraction, and risk of bias assessment were conducted by two authors, independently. A total of 3037 mother-infant pairs from eight randomized controlled trials were ultimately analyzed. The primary outcome was the risk of “asthma and/or wheeze”, and the secondary outcome was “Allergic asthma” in this dose-response meta-analysis. Sensitivity analysis and subgroup analysis were conducted. The robust-error meta-regression model was used for dose-response analysis.

**Results:**

This meta-analysis showed that n-3 LC-PUFA during pregnancy did not obviously reduce the risk of asthma/wheeze (RR 0.93; 95% CI 0.82 to 1.04, *p* = 0.21) and allergic asthma (RR 0.66, 95% CI 0.24 to 1.86, *p* = 0.44). The risk of asthma/wheeze in offspring was significantly decreased in the subgroup analysis when:: (1) studies conducted in Europe (RR 0.69; 95% CI 0.53 to 0.89); (2) daily supplementary dose of n-3 LC-PUFA was at least 1200 mg (RR 0.69; 95% CI 0.55 to 0.88); (3) supplementation lasts from pregnancy to lactation period (RR 0.69; 95% CI 0.51 to 0.95). Furthermore, the risk of asthma/wheeze reduce 2% when daily supplemental dose of n-3 LC-PUFA was increased by 100 mg in the linear dose-response analysis model.

**Conclusions:**

Perinatal supplementation with n-3 LC-PUFA can reduce the incidence of asthma/wheeze and allergic asthma in children under certain conditions, and higher doses indicate better protective effects. Further studies are required to confirm the hypothesis of an association between n-3 LC-PUFA intake and childhood asthma/wheeze prevention.

**Supplementary Information:**

The online version contains supplementary material available at 10.1186/s12887-022-03421-z.

## Background

The prevalence of asthma, the most common allergic disease in childhood, has increased rapidly over the past 20–30 years [1–4]. Children with asthma have compromised life satisfaction, not only in limited physical activity but also in impaired emotional and mental health [5–9]. These negative effects create enormous burdens on families, society, and medical care systems.

Long-chain polyunsaturated fatty acids (LC-PUFAs) refer to fatty acids with two or more unsaturated double bonds and a carbon chain length of at least 20 in the molecular structure [10, 11]. LC-PUFAs typically include omega-3 (n-3), omega-6 (n-6), and omega-9 (n-9) polyunsaturated fatty acids (PUFAs), whose names depend on the position of the first unsaturated carbon double bond, at the third, the sixth, and the ninth carbon of the methyl end [12]. Among them, n-3 and n-6 PUFAs possess important physiological functions [13, 14]. The change of the n-3/n-6 LC-PUFA ratio in the diet, especially the increased consumption of n-6 LC-PUFA and the decreased consumption of n-3 LC-PUFA, are considered to be linked with an increased risk of asthma or wheeze [15–17]. In many countries, high consumption of vegetable oils and meat has increased the intake of n-6 LC-PUFA and arachidonic acid (AA, 20:4, n-6), respectively. Diets with high n-6 LC-PUFA lead to high concentrations of AA in tissues. Additionally, AA produces prostaglandins and leukotrienes, both highly active mediators of inflammation and allergic reactions [18]. In contrast, n-3 LC-PUFA, mainly composed of eicosapentaenoic acid (EPA) and docosahexaenoic acid (DHA), has multiple anti-inflammatory actions. For example, it can reduce leukocyte chemotaxis, adhesion molecule expression and leucocyte-endothelium interaction; decrease the production of inflammatory cytokines and the reactivity of T-cell; increase the production of eicosanoids with relatively low biological potency and inflammation resolving resolvins from EPA and docosahexaenoic acid DHA [19].

Some evidence from observational studies suggested a beneficial effect of n-3 PUFA intake during pregnancy on the risk of allergic diseases, especially asthma/wheeze [20–25]. An observational study by Salam in 2005 revealed that maternal intake of oily fish during pregnancy might be protective against asthma in offspring [21]. In 2007, Willer et al. reported that maternal fish consumption during pregnancy was beneficially associated with allergic diseases, especially childhood asthma [23]. A protective effect of maternal fish intake during pregnancy on the risk of persistent wheeze was observed in many studies [20, 22, 24, 25]. Since it’s well known that fish rich in n-3 LC-PUFAs, many randomized controlled trials (RCTs) were conducted to study the effect of daily intake of DHA or EPA capsules on the risk of asthma/wheeze, and different results were drawn [26–31]. In 2016, Bisgaard et al. reported a significant association between the supplementation of n-3 LC-PUFA and the risk of persistent wheeze or asthma through a study on 695 pregnant women and their infants [26]. In a study published by Hansen in 2017, maternal supplementation with n-3 LC-PUFA might have the prophylactic potential for long-term prevention of asthma or allergic asthma in offspring [27]. These findings, together with observations from other clinical studies [28–31], led to a hypothesis that n-3 LC-PUFA supplementation during pregnancy might affect the risk of asthma/wheeze in offspring.

Many systematic reviews and meta-analyses of RCTs have been performed to obtain better clinical evidence [32–34].. In two studies, the association between maternal n-3 LC-PUFA intake and the risk of allergic diseases in offspring was evaluated [32, 33], but asthma/wheeze was not adequately analyzed. In a systematic review, Lin et al. [34] found a protective effect of prenatal fish oil replenishment on children with wheeze/asthma, while the RCTs retrieved and included in their meta-analysis were not comprehensive.

In this report, we conducted an updated systematic review to further measure a possible relationship between n-3 LC-PUFA supplementation during pregnancy and the risk of asthma/wheeze in offspring, thereby improving the life quality of children with asthma/wheeze.

## Methods

This systematic review of RCTs was performed according to the Preferred Reporting Items for Systematic Reviews and Meta-Analyses (PRISMA) guidelines [35], which reported maternal fish oil intake during pregnancy and asthma/wheeze in children.

### Search strategy

A structured and comprehensive literature search was executed from origin to 31 March 2021 using the following databases: PubMed, Medline, Web of Science, Embase, and the Cochrane library. We searched for relevant publications using the specific search criteria for each database based on PubMed search criteria. The search terms in our search strategy were: ((((((((((fatty acid) OR (omega 3 fatty acid)) OR (n-3 PUFA)) OR (n-3 fatty acid)) OR (n-3 polyunsaturated fatty acid)) OR (docosahexaenoic acid)) OR (fish oil)) OR (fish)) AND ((((((pregnant) OR (perinatal)) OR (prenatal)) OR (antenatal)) OR (maternal)) OR (gestational))) AND (((((child) OR (offspring)) OR (infant)) OR (adolescent)) OR (youth))) AND ((((((asthma) OR (wheeze)) OR (respiratory)) OR (Immunoglobulin E-mediated hypersensitivity)) OR (atopic)) OR (allergic)).

### Study selection

The meta-analysis included studies that met the following criteria: (1) Design: the RCT; (2) Participants: pregnant women and their children; (3) Experimental group: supplement with capsules rich in n-3 LC-PUFA or salmon during pregnancy; (4) Control group: supplement with placebo (e.g., olive oil, soya bean oil, and vegetable oil); (5) Outcomes: prevalence of asthma/wheeze and allergic asthma.

Two authors independently assessed articles for inclusion. Any discrepancies were resolved by discussion and, if necessary, by third-party arbitration.

### Outcome measures

For this review, the primary outcome was the prevalence of asthma/wheeze, defined as a clinical diagnosis, parental report of asthma symptoms, at least three instances of wheeze in the previous 2 years, or parental report of a physician diagnosis of asthma.

The secondary outcome was the prevalence of allergic asthma, defined as asthma with IgE antibodies or a positive skin-prick test.

### Data extraction and Bias assessment

Data were extracted using a standardized table. Extracted data included: first author, year of publication, study location, study design, participants, intervention, placebo, follow-up time, and incidences of asthma/wheeze and allergic asthma.

The quality of studies was assessed using the Cochrane Collaboration Risk of Bias Tool [36]. Two authors indenpendently extracted the data from the selected studies and assessed the quality of the articles. Any differences or discrepancies would be settled through a third-party discussion.

### Data synthesis and analysis

Relative risk (RR) with a 95% confidence interval (CI) was employed to assess the effects of n-3 LC-PUFA supplementation during pregnancy on asthma/wheeze or allergic asthma in offspring. Hazard ratio (HR) and incidence rate ratio (IRR) were directly regarded as the RR. The odds ratio about allergic asthma reported by Hansen et al. was considered the RR due to the lack of details [27]. When RR was not reported in the article, we calculated the crude RR according to the events/total of included studies.

Additionally, *I*^*2*^ statistics were employed for quantifying the potential variability among the included studies. When the heterogeneity was not significant (*I*^*2*^ **≤** 50%), the fixed-effect model was implemented to summarize the results. When the heterogeneity was significant (*I*^*2*^ > 50%), the meta-analysis was performed by a random effect model [37].

Sensitivity analysis was conducted to examine how deletion of a study affects overall results by omitting one trials in turn and recalculating the pooled RRs of the incidence of outcomes. Dose-response data using the robust-error meta-regression method was implemented by Stata 15.1/SE. The Egger test was conducted in this meta-analysis to evaluate potential publication bias. A funnel plot was not executed because the studies were less than ten.

The above analyses and forest plots contained in this review were performed by Stata 15.1/SE. The “risk of bias graph” was created by Review Manager 5.3.

## Results

### Literature search

**Fig. 1:**
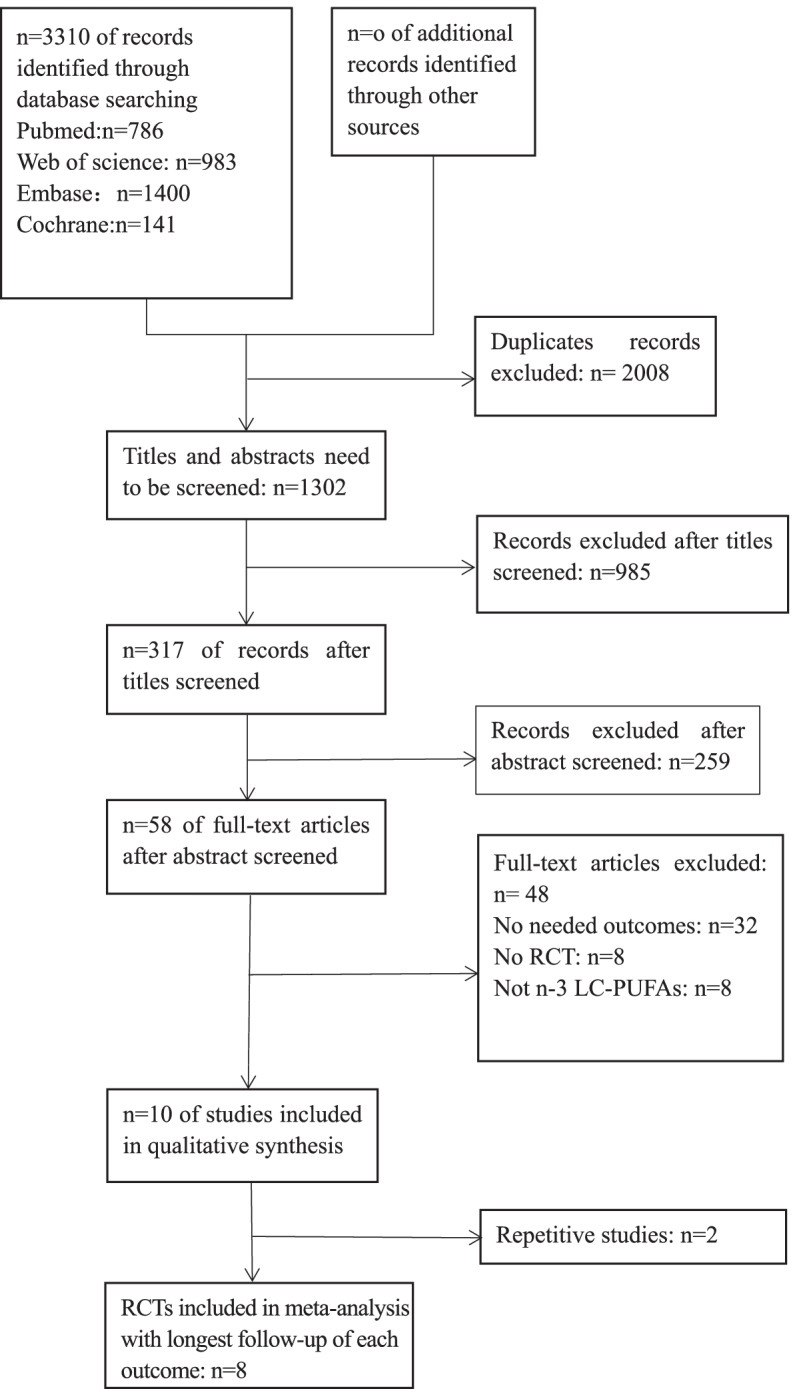
Flow diagram depicting the study selection and progress of RCTs identified in the systematic review and meta-analysis. RCT, randomized controlled trial

Through an online database search, 3310 publications were initially identified from PubMed, Medline, Web of Science, Embase, and the Cochrane library. After removing duplicate articles, 1302 publications remained. Then, 985 publications were removed after reading the titles, and 259 publications were removed after reading the abstracts by two independent reviewers; this process resulted in 58 publications being read in full. In addition, 48 full-text publications were rejected for various reasons, 32 articles did not report the outcomes we needed; 8 articles were observational studies rather than RCTs; and 8 articles were not supplemented with n-3 LC-PUFA. Finally, 10 studies included in the qualitative synthesis, and 8 unique studies with longest follow-up of each outcome included in meta-analysis. A flow diagram of the study selection and progress of RCTs was elaborated in Fig. 1.

### Participants

The data of this review were from 3037 mother-infant pairs, who completed the RCTs. A total of 1605 women were in the experimental group, and 1432 women were in the control group. All participants were recruited from clinics and hospitals. The selected studies were sourced worldwide, with three studies in Australia [26, 38, 39], two RCTs from North America [31, 40], and five studies in Europe [26, 27, 29, 30, 41].

Infant at high risk of allergic disease means one or more fist-degree relatives of him has a history of medically diagnosed allergic diseases. Included infants in four RCTs [28–30, 38] had high risk of allergic diseases. Two trials [26, 27] included pregnant women without diagnosed allergic problems and one trial included women with depression [31]. One RCT included atopic and non-atopic mothers [40]. Pregnant women with atopic disease in this RCT were included in the subgroup of “high infant risk of allergic disease” for analysis. Meanwhile, non-atopic women in this study were included in the subgroup of “non-high infant risk of allergic disease” for analysis. Follow-up time ranged from 6 months to 24 years.

Of the 10 studies included, the studies of Palmer 2013 [39] and Best 2016 [38] are from the same RCT with different follow-up time and the studies of Olsen 2008 [41] and Hansen 2017 [27] are from the same population with different follow-up time, resulting in 8 unique RCTs included in this meta-analysis. The characteristics and results of these RCTs are present in Table 1.

**Table 1:** RCTs of maternal n–3 PUFA supplementation during pregnancy and allergic disease in the offspring

Fist author, country, year	Setting and participants	Intervention and timing	outcomes	Follow-up (age; completed/enrolled, n; rate, %)	Results
Dunstan et al. Australia, 2003	*n* = 98 pregnant, atopic women, were recruited between January 1999 and September 2001 in Western Australia.	Fish oil group (*n* = 52): fish oil capsules 4 × 1 g/d; 3700 mg n-3 LCPUFA (56.0% DHA, and 27.7% EPA) The control group (*n* = 46): olive oil capsule 4 × 1 g /d. From 20 weeks GA until delivery.	Recurrent wheeze.	1 year; 83/98;84.7%	No difference was seen in the incidence of recurrent wheeze (RR,0.90; 95% CI, 0.44 to 1.84; NS).
Olsen et al. Denmark, 2008	*n* = 533 Danish pregnant women with singleton pregnancies through antenatal care clinics in 1990.	Fish oil capsules (*n* = 266), 4 × 1 g/d (32% EPA, 23% DHA). Control 1 (*n* = 136): olive oil capsules. Control 2 (*n* = 131): no oil capsules From 30 weeks GA to delivery.	Asthma of any types; allergic asthma	16 years; 528/533; 99.1%	Asthma (any type) was significantly reduced in the fish oil group compared with the olive oil group (HR,0.37; 95% CI, 0.15–0.92; *P* = 0.03). There was a significant effect on allergic asthma (HR,0.13; 95% CI, 0.03–0.60; *P* = 0.01).
Fist author, country, year	Setting and participants	Intervention and timing	outcomes	Follow-up (age; completed/enrolled, n; rate, %)	Results
Furuhjelm et al., Sweden, 2011	*N* = 145 pregnant women, at risk of having an allergic infant, were recruited through antenatal care clinics in 2003–2005, in Sweden.	The −3 group (*n* = 70): nine capsules (35% EPA, 1.6 g/day and 25% DHA, 1.1 g/day). The placebo group (*n* = 75): Nine soya bean oil capsules From 25 weeks GA to 3.5 months of breastfeeding.	Any asthma; IgE-associated asthma.	2 years, 143/145;98.6%	No difference in the prevalence of any asthma (RR,1.05; 95% CI, 0.41–2.72; NS). and IgE-associated asthma (RR,0.59; 95% CI, 0.11–3.11; NS) between the intervention group and the placebo.
Noakes et al., United Kingdom, 2012	*N* = 123 pregnant women in the area of Princess Anne Hospital (Southampton, United Kingdom) were enrolled in the study.	The salmon group (*n* = 62):2 portions per week of farmed salmon contained 1.14 g EPA, 2.32 g DHA. The control group (*n* = 61): continue their habitual diet. From 20 weeks GA until delivery.	wheeze	0.5 year; 86/123;69.9%	No significant differences in the incidence of wheeze were observed between the groups (RR,1.26; 95% CI, 0.54–2.94; NS).
Fist author, country, year	Setting and participants	Intervention and timing	outcomes	Follow-up (age; completed/enrolled, n; rate, %)	Results
Palmer et al., Australia, 2013	*N* = 706 infants, at high hereditary risk of developing allergic Disease, whose mothers were participating in the DOMInO trial, were recruited from 20th March 2006 and to 8th May 2008, in South Australia.	The n-3LCPUFA group (*n* = 368): 3 × 500 mg capsules/d，800mgDHA,100mgEPA. The control group (*n* = 338): 3 × 500 mg vegetable oil capsules From 21 weeks GA to birth.	IgE-associated asthma	3 years; 638/706;90.4%	No significant differences were seen in the incidence of IgE-associated asthma (RR,0.0.39; 95% CI, 0.15–1.01; NS).
Escamilla-Nuñez et al., Mexico, 2014	*N* = 1094 pregnant women were recruited between February 2005 and February 2007, in Mexico. 586 non-atopic mothers and 283 atopic mothers complete the trial.	DHA group (n = 547): 2capsules/d, contained 400 mg DHA/d. Placebo group (*n* = 547): 2capsules/d, contained a mixture of corn and soy oil. From 18 to 22 weeks GA to delivery.	Wheezing	1.5 years; 869/1094;79.4%	No statistically significant protective effect of DHA treatment in the offspring of maternal atopic, compared with the placebo group was observed on the incidence of wheeze (IRR,0.88; 95% CI, 0.40 to 1.94; *P* = 0.42), and the same to the offspring whose mothers are non-atopic (IRR,1.03; 95% CI, 0.83 to 1.27; *P* = 0.80). And the combined RR of wheeze in the offspring was 0.98 (95% CI, 0.82 to 1.17, NS), whether or not the maternal atopic history.
Fist author, country, year	Setting and participants	Intervention and timing	outcomes	Follow-up (age; completed/enrolled, n; rate, %)	Results
Berman et al., United States, 2016	*N* = 118 women, both with and without history of allergic disease, were recruited in the United States.	Group 1 (*n* = 39): EPA-rich fish oil (1060 mg EPA plus 274 mg DHA). Group 2 (*n* = 38): DHA-rich fish oil (900 mg DHA plus 180 mg EPA). The placebo (*n* = 41): soy oil. From 12 to 20 weeks GA to delivery.	Asthma/wheezing	3 years; 84/118;71.2%	No significant differences were seen in the incidence of asthma/wheeze in the offspring between the fish oil group and the placebo group (RR,0.88; 95% CI, 0.40 to 1.94; NS).
Best et al., Australia, 2016	*N* = 706 children, born to mothers who participated in the (DOMInO) RCT, with a family history of allergic disease, were recruited in South Australia.	The n-3 LCPUFA group (*n* = 368): 500 mg fish oil capsules (800 mg DHA/d, and 100mgEPA/d). Control (*n* = 338): 500 mg vegetable oil capsule From 21 weeks GA until birth.	Wheeze symptoms with sensitization; `parent-reported asthma ever	6 years; 603/706; 85.4%	There was no difference between the n-3 LC-PUFA and control groups in the percentage of children with parent-reported asthma (RR,1.09; 95% CI, 0.86 to 1.39; *P* = 0.92). No significant differences were seen in the incidence of wheeze symptoms with sensitization in the offspring between the groups (RR, 1.22; 95% CI, 0.85 to 1.75; *P* = 0.35).
Fist author, country, year	Setting and participants	Intervention and timing	outcomes	Follow-up (age; completed/enrolled, n; rate, %)	Results
Bisgaard et al., Denmark, 2016	*N* = 695 pregnant women completed the Copenhagen Prospective Studies on Asthma in Childhood2010 (COPSAC2010) pregnancy Cohort, between November 2008 and November 2010.	Fish oil capsules (*n* = 322), 4 × 1 g/d; 2.4 g/d *n* − 3 LCPUFA (55% EPA and 37% DHA); the control group (*n* = 325): olive oil. From 24 weeks GA until 1 week after delivery.	Persistent wheeze or asthma.	6 years; 647/695; 93.1%	The risk of persistent wheeze or asthma in the treatment group was 19.0% vs. 29.2% in the control group (HR,0.66; 95% CI, 0.47 to 0.91; *P* = 0.011).
Hansen et al., Denmark, 2017	*n* = 533 Danish pregnant women with singleton pregnancies through antenatal care clinics in 1990.	Fish oil capsules (*n* = 266), 4 × 1 g/d (32% EPA, 23% DHA). Control 1 (n = 136): olive oil capsules Control 2 (*n* = 131): no oil capsules From 30 weeks GA to delivery.	Asthma medication used; allergic asthma.	2 years; 522/533; 98.0%	Asthma medication prescribed was significantly reduced in the fish oil group compared with the olive oil group (HR,0.54; 95% CI, 0.32–0.90; *P* = 0.02). There was a significant effect on allergic asthma (OR,0.27; 95% CI, 0.08–0.91; *P* = 0.03) between the fish oil group and the olive oil group.

*RCT* Randomized controlled trial, n*-3-PUFA* n-3 polyunsaturated fatty acid, *GA* Gestational age, *RR* Risk ratio, *CI* Confidence interval, *NS* No significance, *HR* Hazard ratio, *IRR* Incidence rate ratio

### Intervention

In six of the eight unique RCTs, participants were divided into an experimental group and a control group [26, 28–30, 38–40]. The experimental group received salmon or fish oil, and the control group received olive oil, vegetable oil, corn and/or soy oils, or nothing. In two trials [27, 31], participants were divided into three groups. In one trial [31], an EPA-rich fish oil group, a DHA-rich fish oil group, and a control group (soy oil) were constituted. Another trial included [27] one experimental group (fish oil) and two control groups (olive oil and no oil). Daily supplementation of n-3 LC-PUFA ranged from 400 mg to 3700 mg.

In six trials, n-3 LC-PUFA supplementation was commenced at 12 to 30 weeks of gestational ages and continued until delivery [27, 28, 30, 31, 38, 39]; in two trials, supplementation continued into the lactation period [26–29]. The duration of intervention was from 10 to 29 weeks after statistical calculation.

### Quality of RCTs

A bias risk assessment was performed using the modified models of the Cochrane Collaboration Risk of Bias Tool for intervention trials for the following six aspects: selection bias, performance bias, measurement bias, attrition bias, reporting bias, and other biases [36]. Individual item was assessed as low-risk, high-risk, or unclear (not given).

**Fig. 2:**
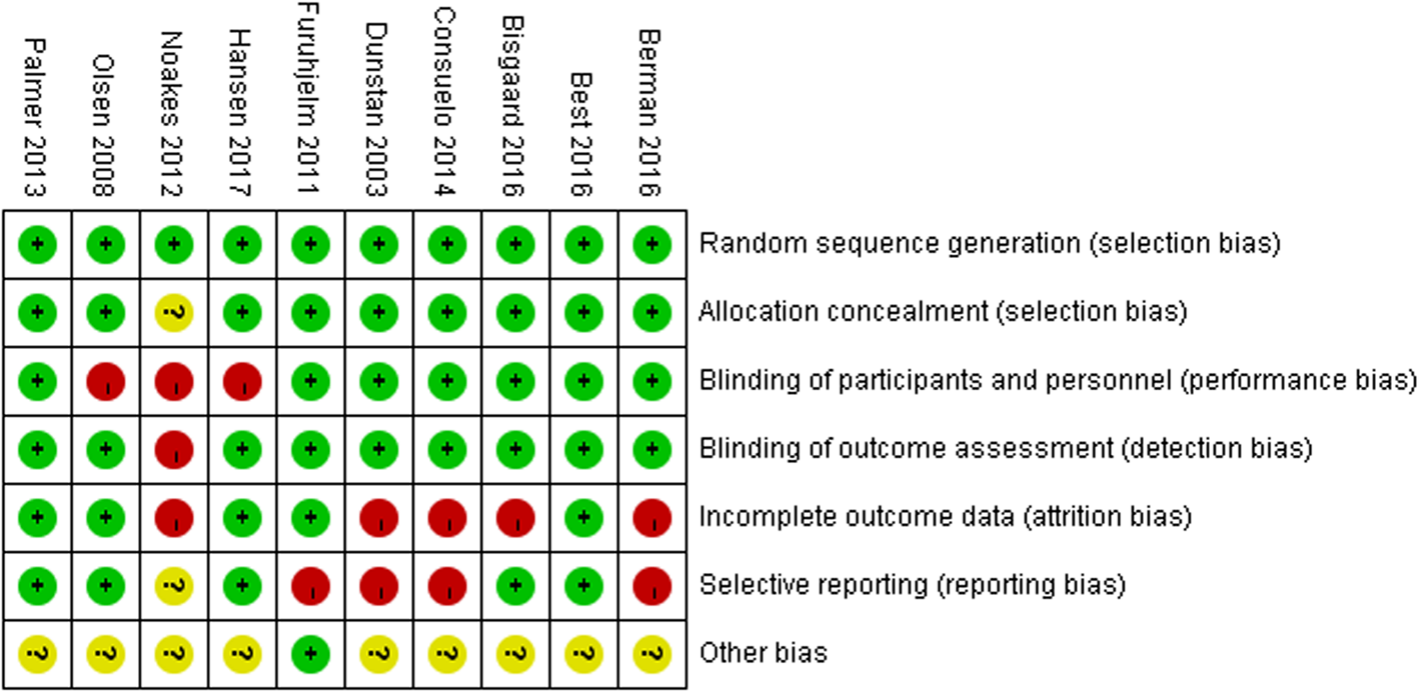
Assessment of risk of bias for included RCTs

The generation of random sequences was assessed as low-risk in all studies. Nine studies [26–29, 31, 38–41] showed ample allocation concealment, and one study [30] did not describe allocation concealment. Thus, experimental subjects and researchers were unable to predict the study results. The pregnant women and medical staff were blinded in seven studies and unblinded in three studies. In the unblinded studies, the pregnant women in the control group did not receive any supplements [27, 30, 41]. Outcome assessors, investigators, and researchers were blinded in all studies. Five studies clearly explained the data, while others did not [26, 28, 30, 31, 39]. Five studies [26, 27, 38, 40, 41] showed a low risk of reporting bias, while four showed a high risk of reporting bias [28, 29, 31, 39]. In nine studies, there was insufficient information to evaluate whether there were other risks of bias or whether current problems introduced bias. Only one study was rated as a low risk of other biases because of no obvious biases in the report [29]. Figure 2 shows the risk assessment of bias for all studies.

### Meta-analysis results of the trials

#### Asthma/wheeze

In clinical practice, asthma in children is more difficult to be explicitly diagnosed because of its strict diagnostic criteria than wheeze [42, 43]. Therefore, the prevalence of asthma/wheeze was chosen as the main outcome to evaluate the effect of fish oil intake during pregnancy. Four reports were from two RCTs. Thus, we assessed eight studies with relatively long follow-up times for the incidence of asthma/wheeze.

**Fig. 3:**
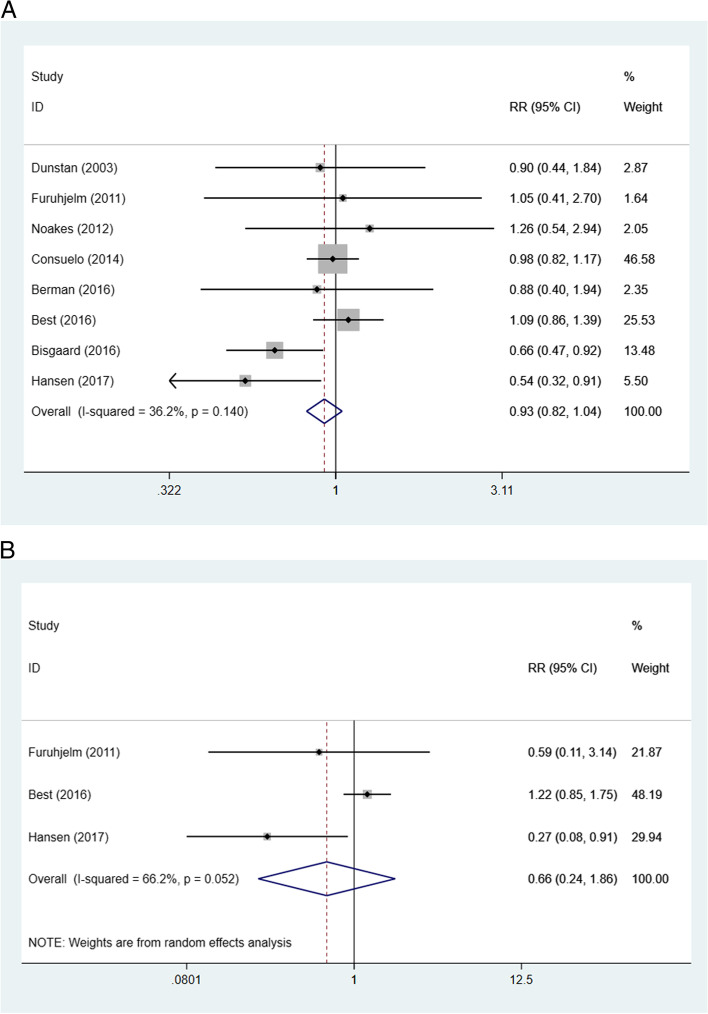
Effect of n-3 LC-PUFA supplementation during pregnancy compared with placebo on the incidence of asthma and/or wheeze (A) and allergic asthma (B) of children. The pooled estimate was obtained using a fixed-effects model depending on the heterogeneity test. Squares represent RRs and error bars represent 95% CIs. The diamond represents the overall effect estimate. The size of the shade square is proportional to the percent weight of each study

Six trials did not reveal any significant differences in asthma/wheeze between the fish oil group and the placebo group [28–31, 38, 39]. Two trials showed significant protective effects of the intervention during pregnancy [26, 27]. The pooled data from eight trials [26–31, 38, 39] showed that n-3 LC-PUFA intake during pregnancy expressed no significant protective effects on asthma/wheeze compared with placebo (RR 0.93; 95% CI 0.82 to 1.04; *p* = 0.21) (Fig. 3**-A**).

#### Allergic asthma

In three RCTs, investigators reported the effect of n-3 LC-PUFA intake on the incidence of allergic asthma [27, 28, 38], with only one trial showing a significant association between maternal fish oil intake and childhood allergic asthma [27]. The pooled data from three trials [27, 28, 38] indicated that the prevalence of allergic asthma in children was not reduced in the experimental group compared with the placebo-controlled group (RR 0.66, 95% CI 0.24 to 1.86, *p* = 0.44**;** Fig. 3**-B)**.

#### Subgroup analysis

The outcomes were analyzed in terms of stratified study location, dose, infant risk of allergic disease, duration of supplementation, and age of offspring.

The risk of our main outcome endpoint in offspring, asthma/wheeze, was significantly decreased when: (1) in Europe; (2) the supplementaion of n-3 LC-PUFA was at least 1200 mg/d; (3) supplementation from pregnancy to lactation. Detailed results of asthma/wheeze subgroup analysis are shown in Table 2. We found that prenatal supplementation with n-3 LC-PUFA could reduce the incidence of allergic asthma in preschool children (6 years of age or younger). Detailed information about the age subgroup of allergic asthma is presented in Table 3.

**Table 2:** Subgroup analyses of n-3 LC-PUFA supplementation during pregnancy on the incidence of asthma/wheeze

Asthma / wheeze	Subgroup classification	Number of studies	RR (95% CI)	P	I^2^ (%)
	Australia [28, 38]	2	1.07 (0.85, 1.34)	0.57	0
Study location	Europe [26, 27, 29, 30]	4	0.69 (0.53, 0.89)	< 0.01	17.4
	North America [31, 40]	2	0.97 (0.82, 1.16)	0.78	0
Dose	≥1200 mg/d [26–29, 31]	5	0.69 (0.55, 0.88)	< 0.01	0
	< 1200 mg/d [30, 38, 40]	3	1.02 (0.89, 1.18)	0.74	0
Infant risk of allergic disease^a^	High [28–30, 38, 40]	5	1.01 (0.85, 1.21)	0.89	0
	Non-high [26, 27, 31, 40]	4	0.86 (0.73, 1.01)	0.07	64
Duration of supplementation	Pregnancy [28–29,31-32,39,41,]	6	0.97 (0.85, 1.11)	0.69	20.8
	Pregnancy and lactation [26, 29]	2	0.69 (0.51, 0.95)	0.02	0
Age of offspring^b^	< 5 years [28–31, 40]	5	0.98 (0.83, 1.16)	0.83	0
	5–18 years [27,39,42,]	3	0.88 (0.73, 1.06)	0.20	78.9
	> 18 years [27]	1	0.54 (0.32, 0.91)	0.02	/

*RR* Risk ratio, *CI* Confidential interval. P is for statistical significance of the subgroup results. I^2^ is for statistical heterogeneity within studies

^a^The subgroup analysis of “Infant risk of allergic disease*” included 8 studies. Pregnant women with atopic disease in Escamilla-Nuñez [40] were included in the subgroup of “high infant risk of allergic disease” for analysis. Meanwhile, non-atopic women in this study [40] were included in the subgroup of “non-high infant risk of allergic disease” for analysis

^b^The subgroup analysis of “Age of offspring” included 9 studies from 8 unique RCTs. The subgroup of “Age of offspring” included nine studies, and two of them were from the same RCT, with result in 16 [41] and 24 years [27] followed up. The study with 16 years follow-up by Olsen [41] were included in the subgroup of “5–18 years” for analysis. Meanwhile, the study with 24 years follow-up by Hansen [27] were included in the subgroup of “> 18 years” for analysis. Therefore, this subgroup included 9 studies from 8 unique RCTs

**Table 3:** Subgroup analyses of n-3 LC-PUFAs supplementation during pregnancy on the incidence of allergic asthma

allergic asthma	Subgroup classification	Number of studies	RR (95% CI)	P	I^2^ (%)
	< 5 years	2	0.43 (0.19, 0.99)	0.047	0
Age of offspring*	5–18 years	2	0.45 (0.05, 3.99)	0.47	87.7
	> 18 years	1	0.27 (0.08, 0.91)	0.04	/

*RR* Risk ratio, *CI* Confidential interval, P is for statistical significance of the subgroup results. I^2^ is for statistical heterogeneity within studies

### Sensitivity analysis

Sensitivity analysis did not show significant changes in asthma/wheeze outcomes, with the pooled RRs ranging from 0.87 (95% CI 0.76–1.01) to 0.98 (95% CI 0.86–1.11). Sensitivity analysis of allergic asthma outcomes showed the pooled RRs ranged from 0.36 (95% CI 0.13–0.95) to 1.18 (95% CI 0.83–1.68). **Appendix** **1****–1 and Appendix** **1****–2** exhibit a detailed record of the sensitivity analysis.

### Dose-response analysis

**Fig. 4:**
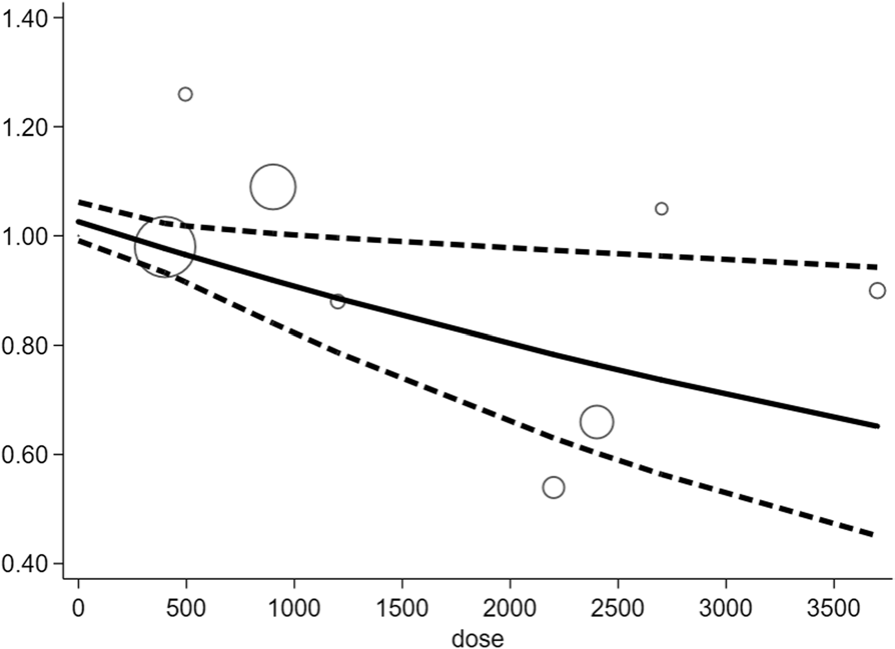
Dose-response regression analysis between daily dose of n-3 LC-PUFA supplement and the risk of asthma/wheeze

In this study, the RMER (robust error meta-regression) model was used for dose-response regression analysis. The *p*-value of the non-linear Chi-square test was 0.3212, so the linear model was chosen for dose analysis. The results showed a linear dose-response relationship between the daily dose of n-3 LC-PUFA supplement during pregnancy and the incidence of asthma/wheeze. Higher doses indicated lower incidence. Moreover, when perinatal n-3 LC-PUFA supplementation was increased by 100 mg/d, the risk of asthma/wheeze was reduced by 2%. Details of the linear regression model of the dose-response analysis are shown in Fig. 4, and other detailed data and code of Stata on dose-response analysis are shown in **Appendix** **2****.**

### Publication bias

The Egger test for asthma/wheeze (*p* = 0.54) and allergic asthma (*p* = 0.34) revealed no statistically significant publication bias for each outcome in this meta-analysis.

## Discussion

The current systematic review did not show an explicit relationship between the prenatal intake of n-3 LC-PUFA and the prevalence of asthma/wheeze in offspring, similar to a recent study by Vahdaninia et al. [33] and summarized in a review by Best et al. [32].

The result of the subgroup analysis was interesting. Studies in Europe showed a protective effect on wheeze/asthma in offspring. After removing a study conducted in Mexico, a large sample trial included in our review (the heterogeneity decreased from 64 to 0%; **Appendix** **3****)** maternal supplementation with fish oil during pregnancy did not show any advantage in preventing asthma/wheeze for children without family histories of allergic diseases (i.e., medically diagnosed allergic diseases, e.g., eczema, asthma, or hay fever),**.** We attributed those changes to differences in ethnicity and environment. Moreover, the association between the prevalence of asthma and race/ethnicity has also been confirmed in other studies [44–47]. In addition, it is speculated that the ineffective fish oil supplementation during pregnancy in some countries or regions may be related to the high baseline levels of n-3 in the population. However, in current RCTs, the reports of the baseline level of n-3 are generally lacking. Thus further RCTs are encouraged to explore the issues.

Compared with the effective dose of 2000 mg/d reported in a previous review [34], the dose-response curve in this study suggested that only 1200 mg DHA and/or EPA per day can significantly prevent asthma/wheeze. The dosage of drugs is very important for treating diseases [48, 49]. Different dosages of the same drug exert different effects, and the same is true for the supplement of n-3 LC-PUFA. It is reasonable to speculate that intake of very low doses of n-3 LC-PUFA may be meaningless, while supplementation with high doses may increase the risk of fatty liver and even muscle damage [50, 51]. Given the hazards of excessive doses of fish oil supplements, the upper limit of fish oil supplementation requires further investigation in depth.

Supplementation with fish oil from 22nd weeks of gestational age to early lactation significantly reduced the prevalence of asthma/wheeze in offspring. We hypothesized that n-3 LC-PUFA might have different activities in embryo development at different stages of pregnancy because pro-inflammatory immune cell genes are expressed in late pregnancy, and the period of late pregnancy has a critical regulatory function in inflammatory and immune system development [52, 53]. The immune system of a newborn is highly malleable. If the immune system does not receive appropriate signals, newborns will be susceptible to allergic diseases [54–56]. Therefore, we speculated that there is a “window of opportunity” in early life during which the immune system may be influenced by fish oil to limit susceptibility to allergic diseases.

Atopic march was considered as a progression and accumulation of atopic conditions an individual (usually a child) gets older, characterized by the onset and resolving of symptoms of allergic disease over time [57]. Asthma symptoms among adults may originate in childhood [58]. Phenotypes of asthma in children are commonly associated with allergy, and the incidence of allergic asthma declines with age [59–61]. Studies have suggested that early-onset asthma can be attributed to genetic, epigenetic and atopic factors, while late-onset asthma may be related to environmental risk factors [58, 62]. The subgroup analysis revealed that fish oil supplementation reduced the prevalence of allergic asthma in preschool children (< 6 years). However, underlying molecular mechanisms for the associations remain unclear. More studies are needed to measure the relationship between the maternal fish oil supplement and allergic asthma in childhood through both laboratory and epidemiological cohort studies.

Our meta-analysis exhibits several advantages. All included studies are recent RCTs with large sample sizes. The subgroup analysis and sensitivity analysis have been performed to assess potential confounding factors and the stability of the outcomes. The main strength of this systematic review lies in that the most precise and broadest definitions of asthma --"allergic asthma” and “asthma/wheeze” are used as our outcome variables. However, our systematic review has some limitations. The extrapolation of the conclusion needs to be further verified because of the unavoidable risk of multiple analysis and chance finding. The primary studies have different protocols, which may affect our findings. For example, the baseline characteristics of pregnant women, the dosage of intervention, and the diagnosis of outcomes are different.

The hypothesis linking maternal n–3 LC-PUFA intake to protection against childhood asthma/wheeze or allergic asthma cannot be absolutely accepted or rejected due to the lack of new and large-sized RCTs, possible confounding factors, and potential bias.

The race, dosage, susceptibility of n-3 LC-PUFA, and supplementation time should be assessed in the future. Large-sample and multi-center RCTs are required to better comprehend the efficacy of supplementation with n-3 LC-PUFA during pregnancy for protecting against asthma/wheeze or other relevant allergic diseases.

## Conclusion

The results showed that prenatal supplementation with n-3 LC-PUFA may reduce the incidence of asthma/wheeze or allergic asthma in offspring under certain conditions. According to the dose-response analysis, higher doses suggest stronger protective effects. Further high-quality RCTs with large sample sizes should be performed, especially for different races and regions, to examine the effects of reasonable doses of prenatal n-3 PUFA intake and asthma/wheeze in offspring.

## Supplementary Information

**Additional file 1 Appendix 1–1.** Sensitivity analyses of the effect of n-3 PUFA supplementation during pregnancy on the incidence of asthma/wheeze. **Appendix 1–2.** Sensitivity analyses of the effect of n-3 PUFA supplementation during pregnancy on the incidence of allergic asthma.

**Additional file 2 Appendix 2.** Data of dose-response analysis on perinatal supplementation of n-3 PUFA and risk of asthma/wheeze.

**Additional file 3 Appendix 3.** Sensitivity analyses of non-high risk studies on the incidence of asthma/wheeze.

## Data Availability

The datasets analyzed in this study are available from the corresponding author on reasonable request.

## Abbreviations

N-3 LC-PUFA: Omega-3 long-chain polyunsaturated fatty acids
EPA: Eicosapentaenoic acid
DHA: Docosahexaenoic acid
RCTs: Randomized controlled trials
PRISMA: Systematic Reviews and Meta-Analyses
RR: Relative risk
CI: Confidence interval
HR: Hazard ratio
IRR: Incidence rate ratio
