# Supplementary material for: A dose-response meta-analysis of the association between the maternal omega-3 long-chain polyunsaturated fatty acids supplement and risk of asthma/wheeze in offspring
Source: BMC Pediatr. 2022 Jul 16;22:422. doi: 10.1186/s12887-022-03421-z (PMC9287871; doi:10.1186/s12887-022-03421-z)
Supplement: Supplementary file 1 — Additional file 1 Appendix 1–1. Sensitivity analyses of the effect of n-3 PUFA supplementation during pregnancy on the incidence of asthma/wheeze. Appendix 1–2. Sensitivity analyses of the effect of n-3 PUFA supplementation during pregnancy on the incidence of allergic asthma. [file 12887_2022_3421_MOESM1_ESM.docx]

**Appendix 1-1.** Sensitivity analyses of the effect of n-3 PUFA supplementation during pregnancy on the incidence of asthma/wheeze.

| Study omitted | RR (95%CI) | *I^2^* |
| --- | --- | --- |
| Dunstan (2003) | 0.93 (0.82, 1.05) | 47% |
| Furuhjelm (2011) | 0.92 (0.82, 1.04) | 46% |
| Noakes (2012) | 0.92 (0.81, 1.04) | 44% |
| Escamilla-Nuñez (2014) | 0.88 (0.75, 1.04) | 43% |
| Berman (2016) | 0.93 (0.82, 1.05) | 47% |
| Best (2016) | 0.87 (0.76, 1.01) | 32% |
| Bisgaard (2016) | 0.98 (0.86, 1.11) | 8% |
| Hansen (2017) | 0.95 (0.84, 1.08) | 11% |

RR: relative risk; CI: confidential interval. I^2^ is for statistical heterogeneity of the other studies.

**Appendix 1-2.** Sensitivity analyses of the effect of n-3 PUFA supplementation during pregnancy on the incidence of allergic asthma.

| Study omitted | RR (95%CI) | *I^2^* |
| --- | --- | --- |
| Furuhjelm (2011) | 0.64 (0.15, 2.78) | 82% |
| Best (2016) | 0.36 (0.13, 0.95) | 0% |
| Hansen (2017) | 1.18 (0.83, 1.68) | 0% |

RR: relative risk; CI: confidential interval. I^2^ is for statistical heterogeneity of the other studies.
