# Supplementary material for: A dose-response meta-analysis of the association between the maternal omega-3 long-chain polyunsaturated fatty acids supplement and risk of asthma/wheeze in offspring
Source: BMC Pediatr. 2022 Jul 16;22:422. doi: 10.1186/s12887-022-03421-z (PMC9287871; doi:10.1186/s12887-022-03421-z)
Supplement: Supplementary file 2 — Additional file 2 Appendix 2. Data of dose-response analysis on perinatal supplementation of n-3 PUFA and risk of asthma/wheeze. [file 12887_2022_3421_MOESM2_ESM.docx]

**Appendix 2.** Data of dose-response analysis on perinatal supplementation of n-3 PUFA and risk of asthma/wheeze.

| id | study | cat | year | RR | LCI | UCI | method |
| --- | --- | --- | --- | --- | --- | --- | --- |
| 1 | Dunstan | 0 | 2003 | 1 | 1 | 1 | 0 |
| 1 | Dunstan | 1 | 2003 | 0.90 | 0.44 | 1.84 | 3700 |
| 2 | Furuhjelm | 0 | 2011 | 1 | 1 | 1 | 0 |
| 2 | Furuhjelm | 1 | 2011 | 1.05 | 0.41 | 2.7 | 2700 |
| 3 | Noakes | 0 | 2012 | 1 | 1 | 1 | 0 |
| 3 | Noakes | 1 | 2012 | 1.26 | 0.54 | 2.94 | 494 |
| 4 | Escamilla-Nuñez | 0 | 2014 | 1 | 1 | 1 | 0 |
| 4 | Escamilla-Nuñez | 1 | 2014 | 0.98 | 0.82 | 1.17 | 400 |
| 5 | Berman | 0 | 2016 | 1 | 1 | 1 | 0 |
| 5 | Berman | 1 | 2016 | 0.88 | 0.4 | 1.94 | 1200 |
| 6 | Best | 0 | 2016 | 1 | 1 | 1 | 0 |
| 6 | Best | 1 | 2016 | 1.09 | 0.86 | 1.39 | 900 |
| 7 | Bisgaard | 0 | 2016 | 1 | 1 | 1 | 0 |
| 7 | Bisgaard | 1 | 2016 | 0.66 | 0.47 | 0.92 | 2400 |
| 8 | Hansen | 0 | 2017 | 1 | 1 | 1 | 0 |
| 8 | Hansen | 1 | 2017 | 0.54 | 0.32 | 0.91 | 2200 |

**Code of dose-response analysis in Stata**

#Noninear Model

ssc install remr

findit xblc

remr lnRR selnRR method, id(id) cat(cat) rcs(3) table bplot eform

#Nonlinear test

test __doses1=__doses2

#Linear Model

remr lnRR selnRR method, id(id) cat(cat) table bplot eform
