# Supplementary material for: A dose-response meta-analysis of the association between the maternal omega-3 long-chain polyunsaturated fatty acids supplement and risk of asthma/wheeze in offspring
Source: BMC Pediatr. 2022 Jul 16;22:422. doi: 10.1186/s12887-022-03421-z (PMC9287871; doi:10.1186/s12887-022-03421-z)
Supplement: Supplementary file 3 — Additional file 3 Appendix 3. Sensitivity analyses of non-high risk studies on the incidence of asthma/wheeze. [file 12887_2022_3421_MOESM3_ESM.docx]

**Appendix 3.** Sensitivity analyses of non-high risk studies on the incidence of asthma/wheeze.

| Study omitted | RR (95%CI) | *I^2^* |
| --- | --- | --- |
| Escamilla-Nuñez-2 (2014) | 0.65 (0.50, 0.85) | 0% |
| Berman (2016) | 0.75 (0.51, 1.11) | 75% |
| Bisgaard (2016) | 0.83 (0.55, 1.25) | 56% |
| Hansen (2017) | 0.85 (0.60, 1.19) | 60% |

RR: relative risk; CI: confidential interval. I^2^ is for statistical heterogeneity of the other studies.
